# Highly Reliable Memory Operation of High-Density Three-Terminal Thyristor Random Access Memory

**DOI:** 10.1186/s11671-022-03667-7

**Published:** 2022-02-23

**Authors:** Hyangwoo Kim, Hyeonsu Cho, Hyeon-Tak Kwak, Myunghae Seo, Seungho Lee, Byoung Don Kong, Chang-Ki Baek

**Affiliations:** 1 Department of Convergence IT Engineering and Future IT Innovation Laboratory, Pohang University of Science and Technology (POSTECH), 37673, Pohang, South Korea; 2 Department of Electrical Engineering, Pohang University of Science and Technology (POSTECH), 37673, Pohang, South Korea; 3 Future IT Innovation Laboratory, Pohang University of Science and Technology (POSTECH), 37673, Pohang, South Korea

**Keywords:** Capacitorless 1T DRAM, Memory array operation, Memory disturbance, Three-terminal TRAM, Gated-thyristors

## Abstract

Three-terminal (3-T) thyristor random-access memory is explored for a next-generation high-density nanoscale vertical cross-point array. The effects of standby voltages on the device are thoroughly investigated in terms of gate–cathode voltage (*V*_GC,ST_) and anode–cathode voltage (*V*_AC,ST_) in the standby state for superior data retention characteristics and low-power operation. The device with the optimized *V*_GC,ST_ of − 0.4 V and *V*_AC,ST_ of 0.6 V shows the continuous data retention capability without refresh operation with a low standby current of 1.14 pA. In addition, a memory array operation scheme of 3-T TRAM is proposed to address array disturbance issues. The presented array operation scheme can efficiently minimize program, erase and read disturbances on unselected cells by adjusting gate–cathode voltage. The standby voltage turns out to be beneficial to improve retention characteristics: over 10 s. With the proposed memory array operation, 3-T TRAM can provide excellent data retention characteristics and high-density memory configurations comparable with or surpass conventional dynamic random-access memory (DRAM) technology.

## Introduction

The scaling down of dynamic random-access memory (DRAM) cell has been continuously required for high-density, high-speed, and low-power operations [1–3] However, the conventional *one transistor-one capacitor* (1T- 1C) DRAM is facing an inevitable problem: it is increasingly difficult to achieve the required capacitance to differentiate the two states (~ 10 fF/cell) with the smaller cell area [4]. Even though there have been many studies to improve the capacitor technologies, such as new high-*k* materials [5–7] and a high-aspect-ratio 3D capacitor structure [8, 9], these approaches possess the issue of increasing fabrication complexities and high cost [2, 3]. To overcome these challenges, a capacitorless 1T DRAM structure, namely a *thyristor-based random-access memory* (TRAM), has been proposed as an alternative in which the charge is stored at the internal *p-base and n-base* storage area [10–16]. The TRAM can operate as a two-terminal (2-T) device by modulating the energy band with only the anode and cathode biases [10–12]. 2-T TRAM has the advantage of its simple structure that allows a cost-effective cross-point array fabrication with conventional Si processes. Yet, the drawbacks are the low data retention and array disturbance that stem from the weak controllability and asymmetric of the storage areas [10–12]. The 2-T TRAM has a limit to overcome these drawbacks by controlling the two storage areas with only anode–cathode bias because electrons and holes exhibit a difference in mobility and lifetime. On the other hand, a three-terminal (3-T) TRAM, whose gate bias controls the energy band of the storage region, can remedy these drawbacks; the proper adjustment of gate–cathode voltage (*V*_GC_) can improve the retention characteristics and the array disturbance immunity by anode–cathode voltage (*V*_AC_) [13–16]. But an array operating conditions using the standby voltages for continuous retention characteristics and high disturbance immunity have not been reported yet. In terms of cell density, if a vertical channel transistor (VCT) is adopted, 4F^2^ memory feature size can be achievable with the 3-T TRAM [17, 18].

This paper aims at providing memory operating voltage guidelines with optimized standby voltages in the 3-T TRAM array configurations. The effects of the gate–cathode voltage on the standby state (*V*_GC,ST_) are thoroughly investigated for low-power operations and better retention characteristics. To maintain the stored charges in the storage area with the lowest standby current, a minimum anode–cathode voltage on the standby state (*V*_AC,ST_) is obtained from the anode current–anode voltage characteristics (*I*_A_-*V*_AC_). Furthermore, for a reliable array operation, the operating conditions are suggested to avoid any possible array disturbance using the optimal standby voltage.

## Simulation Methods

**Fig. 1:**
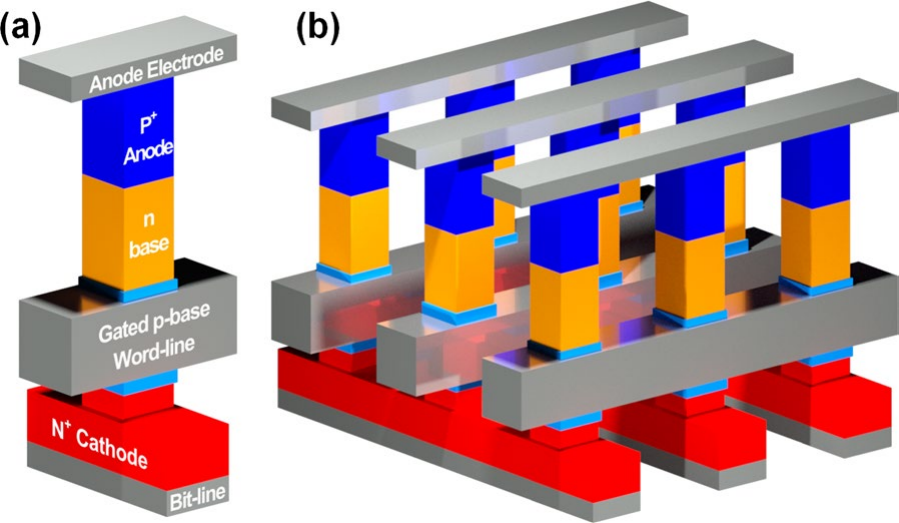
Schematic diagrams of **a** 3-T TRAM unit cell and **b** cross-point vertical 3-T TRAM array

Figure 1 shows a schematic diagram of a 3-T TRAM unit cell and a possible cross-point vertical array configuration. The 3-T TRAM consists of physical *p*^+^-anode—*n*-base—*p*-base—*n*^+^-cathode layers with a gated *p*-base. The anode and cathode areas are highly doped with a doping concentration of 1 × 10^20^ cm^−3^, and the base areas (*p*- and *n*-base) have the same Gaussian doping profile with a peak value of 1.6 × 10^18^ cm^−3^, which have similar doping concentrations in real device. This symmetric doping profile can secure memory hysteresis characteristics and sufficient memory window margin. If the lengths of both *n*- and *p*-bases are shorter than the sum of the depletion width inside the storage areas, sensing margin and storage capacity are reduced. On the other hand, when the length of both bases is longer than the sum of the depletion width, the robustness of the memory structure is worsened due to the high vertical aspect ratio of the 3-T TRAM. Therefore, the lengths of both *n*- and *p*-bases are set to 100 nm considering the junctions' depletion widths [12]. The channel area is 20 × 20 nm^2^, and the thickness of the gate oxide is 5 nm. The gate and cathode electrodes are designed as a word line (WL) and a bit line (BL), respectively.

**Fig. 2:**
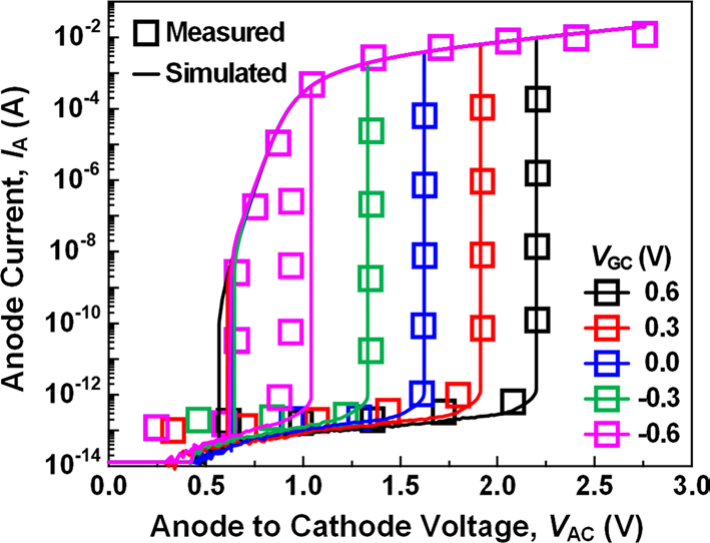
*I*_A_-*V*_AC_ characteristics for various *V*_GC_s simulation results (lines) adjusted to the experimental data (symbols) of the *p*^+^−*n*−*p*−*n*^+^ silicon memory device with a gated base

Si-based 3-T TRAM cells are simulated using Sentaurus technology computer-aided design (TCAD) [19]. To reliably simulate the Si-based 3-T TRAM, the physics models of the simulation are adjusted by using the experimental data of *I*_A_ − *V*_AC_ characteristics for various *V*_GC_s in the Si-based thyristor memory device with a gated base (Fig. 2) [20]. Specifically, the parameters of the recombination model are adjusted and the Si-SiO_2_ surface SRH recombination model is adopted to reflect the data retention characteristics of real devices affected by junction and interface defects. Usual drift–diffusion transport model with Fermi–Dirac distribution is used. Philips unified mobility model is adopted to consider the carrier-impurity and carrier-carrier scatterings [21], and the high-field saturation and doping-dependent mobility models are also used. Oldslotboom bandgap narrowing model [22] is used to consider the highly doped silicon regions. Doping-dependent Shockley–Read–Hall (SRH) [23] and Auger recombination [24] models are adopted to account for the carrier recombination at the junctions. The avalanche generation [25] and band-to-band tunneling [26] models are also considered to calculate the carrier generations and the tunneling. The pulses applied to all memory operations have the rise time (*T*_rise_) and the fall time (*T*_fall_) of 0.25 ns, while the hold time (*T*_hold_) is 2 ns [12]. The operation speed inferred from these pulse parameters is comparable to the modern DRAM memory clock rate [27].

## Results and Discussion

### Optimization of Standby Voltages

**Fig. 3:**
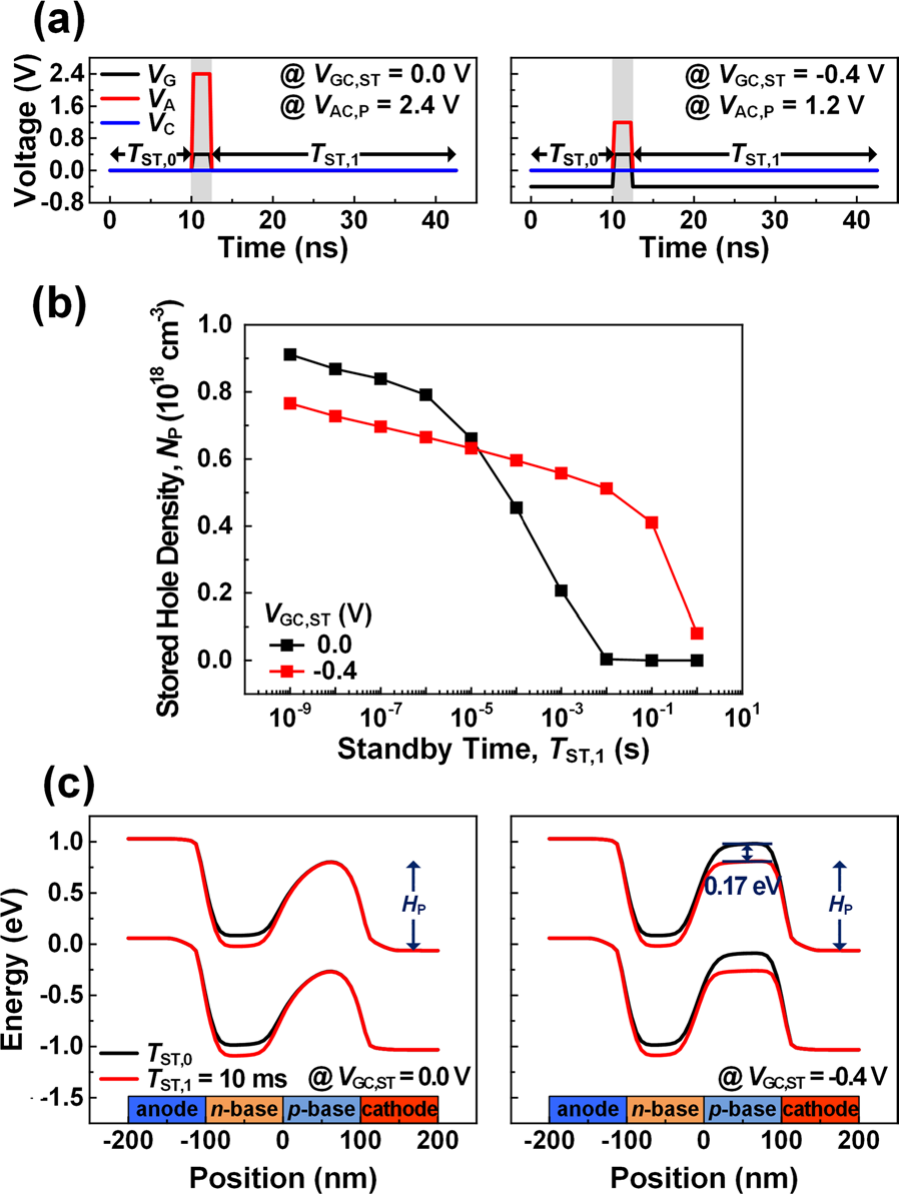
**a** Program operation conditions at a *V*_C_ of 0 V when a *V*_GC,ST_ is 0.0 V (left) and − 0.4 V (right). **b** *N*_P_ as a function of *T*_ST,1_ at different *V*_GC,ST_ of 0.0 V (black line) and − 0.4 V (red line). **c** Energy band diagrams at 10 ms of *T*_ST,1_ when *V*_GC,ST_ is 0.0 V (left) and − 0.4 V (right)

Figure 3a shows two possible approaches to program a cell. In both approaches, the cathode voltage (*V*_C_) is defined as 0.0 V. However, the voltage differences between the gate and the cathode in the standby state (*V*_GC,ST_) are set to two different values. − 0.4 V (right) is the optimized value, while 0.0 V (left) is the conventional case for a comparison. The device with the *V*_GC,ST_ of − 0.4 V has a low program anode–cathode voltage (*V*_AC,P_) of 1.2 V, and this is only a half of *V*_AC,P_ required when *V*_GC,ST_ is 0.0 V. This low *V*_AC,P_ is attributed to the accumulated holes in *p*-base by the negative *V*_GC,ST_. When the *V*_GC_ rapidly rises to 0.4 V for a program operation, the accumulated holes by the negative *V*_GC,ST_ (− 0.4 V) reduce the energy band barrier height in the *p*-base (*H*_P_). As such, the device with the optimized *V*_GC,ST_ minimizes the power consumption in program operation since a smaller *V*_AC,P_ is required to reduce the *H*_P_. Figure 3b shows the stored hole density in the *p*-base (*N*_P_) as a function of standby time at state-1 (*T*_ST,1_) after the program pulse. As the *T*_ST,1_ increases, the *N*_P_ decreases due to the carrier recombinations at the junctions. Due to the low *V*_AC,P_, the device with *V*_GC,ST_ of − 0.4 V exhibits lower *N*_P_ than the case of 0.0 V in the early stage (*T*_ST,1_ < 10 μs). However, the *N*_P_ in the later stage (*T*_ST,1_ > 10 s) is higher than the case of 0.0 V. This higher *N*_P_ is the result of the low recombination rate caused by the depletion of electrons in the *p*-base due to the negative *V*_GC,ST_. Figure 3c shows the energy band diagrams of 3-T TRAM at 10 ms after a program pulse to investigate the data retention characteristic depending on *V*_GC,ST_. The left side is for *V*_GC,ST_ = 0.0 V, and the right side is for *V*_GC,ST_ = − 0.4 V. The *H*_P_ difference between the state-0 and state-1 at 10 ms after a program pulse exhibits a high value of 0.17 eV with the optimized *V*_GC,ST_ of − 0.4 V due to the long-lasting *N*_P_. This indicates that the device with an optimized *V*_GC,ST_ has an improved data retention characteristics that can maintain the low-resistance state (state-1) for a longer time.

**Fig. 4:**
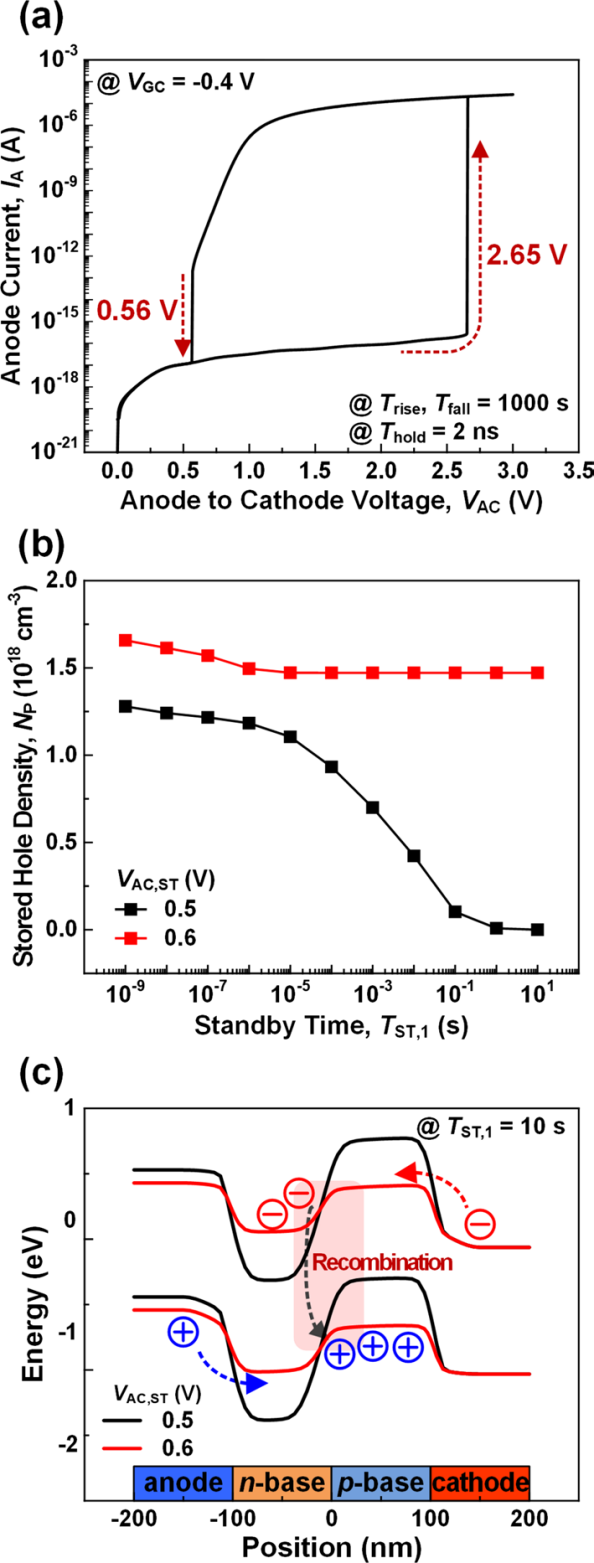
**a** *I*_A_ − *V*_AC_ characteristics of the 3-T TRAM by the *V*_AC_ pulse with *T*_rise_, *T*_fall_ = 1000 s, *T*_hold_ = 2 ns when the *V*_GC_ is fixed at − 0.4 V. **b** *N*_P_ as a function of *T*_ST,1_ at different *V*_AC,ST_ of 0.5 V (black line) and 0.6 V (red line). **c** Energy band diagrams at *T*_ST,1_ = 10 s with different *V*_AC,ST_ of 0.5 V and 0.6 V

Figure 4a shows *I*_A_ − *V*_AC_ characteristics for the *V*_AC_ pulse with *T*_rise_ of 1000 s, *T*_hold_ of 2 ns and *T*_fall_ of 1000 s when *V*_GC_ is fixed at − 0.4 V. It has been previously reported that the *I*_A_ − *V*_AC_ curve with long *T*_fall_ of 1000 s can effectively provide a minimum *V*_AC,ST_ to improve the data retention characteristics [12]. When *V*_GC_ is fixed to − 0.4 V, the device exhibits a rapid increase of *I*_A_ at *V*_AC_ = 2.65 V representing a switching from the state-0 to the state-1. This indicates that a higher *V*_AC_ is required to switch the state as long as *V*_GC_ is maintained below − 0.4 V, and thus the state is well protected. As mentioned above, for a normal programming, only 1.2 V of *V*_AC,P_ is required since *V*_GC_ is increased from − 0.4 to 0.4 V. As such, for *V*_AC,P_ less than 2.6 V applied to the bit line (BL) in array operation, 3-T TRAM can avoid unwanted program errors if the voltage of the word line (WL) is fixed to *V*_GC,ST_ = − 0.4 V or below.

In the downward *V*_AC_ sweep (*T*_fall_), the switching of the state occurs at 0.56 V as evidenced by the sharp slope (red dashed line in Fig. 4a). Thus, the state-1 can be maintained at *V*_AC,ST_ of 0.56 V. To investigate this drastic change by the voltage difference as small as 0.1 V, *N*_P_ as a function of *T*_ST,1_ is examined for two different *V*_AC,ST_ of 0.5 V and 0.6 V (Fig. 4b). For *V*_AC,ST_ = 0.5 V, the stored holes disappear rapidly after 10 μs, but for *V*_AC,ST_ = 0.6 V, the device can maintain a high *N*_P_ of about 1.47 × 10^18^ cm^−3^ for more than 10 s which is 10^6^ times larger. Figure 4c shows the energy band diagrams of 3-T TRAM at *T*_ST,1_ = 10 s for two different *V*_AC,ST_ of 0.5 V and 0.6 V. With *V*_AC,ST_ of 0.6 V, holes more than the amount recombines are injected into the base region, and the state-1 band shape along with the stored charge are maintained. With *V*_AC,ST_ of 0.5 V, on the other hand, the holes injection is not enough to compensate the loss of holes by recombination, and the stored charge rapidly disappears, returning the band shape back to that of the state-0. A device with *V*_AC,ST_ lower than 0.5 V will face similar level or faster charge loss. Considering the clock speed of modern VLSI circuit, the 3-T TRAM with *V*_AC.ST_ of 0.6 V can exhibit the continuous state-1 virtually without a refresh operation. In addition, despite the high *V*_AC,ST_ of 0.6 V, the device has a standby current as low as 1.14 pA, suggesting that the 3-T TRAM with the *V*_AC,ST_ of 0.6 V is suitable for a low-power operation.

### Memory Operation of 3-T TRAM Array

**Fig. 5:**
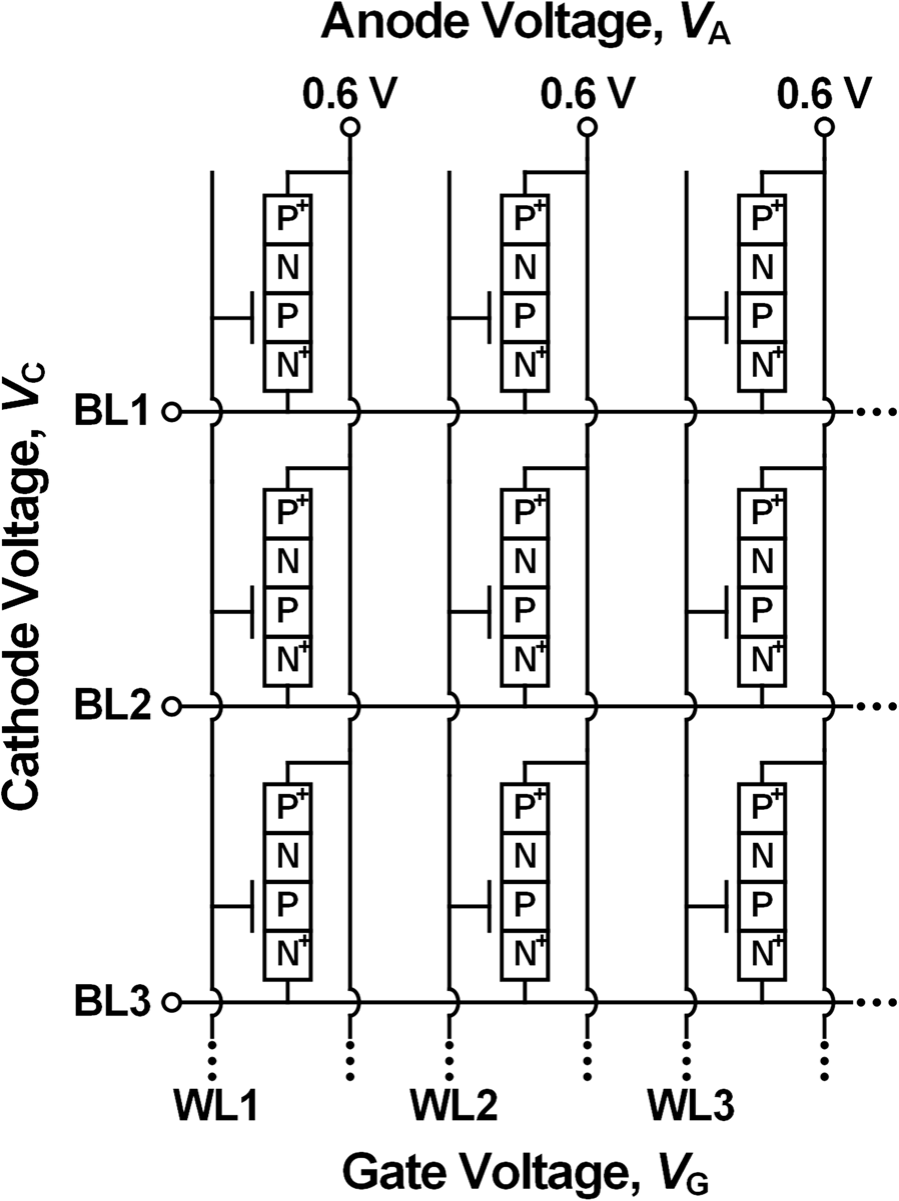
Schematic diagram of the 3-T TRAM memory-cell-array configuration

Compared to the 2-T TRAM without the gate terminal, the 3-T TRAM has a strong state immunity against the change of anode–cathode potential by controlling the storage potential with the gate terminal. On the other hand, the shift in gate–cathode potential in the 3-T TRAM easily interferes with the stored information. This disturbance is studied by assuming an operation pulse applied to a nearby cell. The cell under the study is initially at unselected bias condition, and the subject cell's states after the disturbance are observed. Figure 5 shows the schematic of a memory-cell-array configuration of 3-T TRAM. Our study shows that, with a proper operating scheme (maintaining fixed *V*_GC_ to the unselected cells), this memory-cell-array configuration can prevent unselected cells' unwanted changes. For an efficient adjustment of *V*_GC_ in the memory operation, the gate and cathode electrodes are set to the WL and BL, respectively. The anode electrode is fixed at 0.6 V. Table. 1 shows the operating voltage conditions for the 3-T TRAM array. To maintain the stable state-0 and state-1 in the standby state, *V*_G_ and *V*_C_ in the standby state are set to − 0.4 V and 0.0 V with the *V*_A_ of 0.6 V. The operation strategies to prevent the array disturbance, found through our study, are summarized for each operation (program, erase and parallel read) as the followings.

**Table 1:** Operating voltage condition of 3-T TRAM array

Operation mode	*V*_A_, (V)	Word line (WL), *V*_G_ (V)		Bit line (BL), *V*_C_ (V)	
		Selected	Un-sel	Selected	Un-sel
Standby	0.6	− 0.4		0.0	
Program		− 0.4	− 1.2	− 0.8	0.0
Erase		0.8	− 0.4	0.4	1.2
Read		− 0.8		− 0.8	0.0

**Fig. 6:**
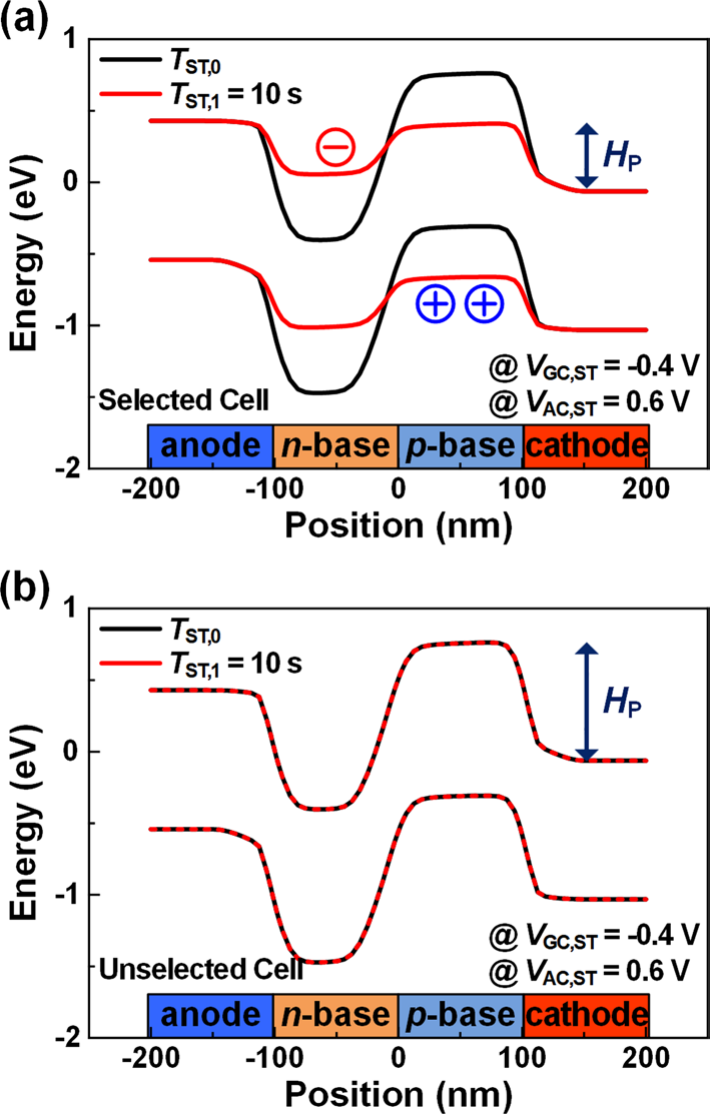
Energy band diagrams of **a** the selected cell for programming at *T*_ST,0_ (black line) and *T*_ST,1_ = 10 s (red line) and **b** that of the unselected cells at *T*_ST,0_ (black line) and *T*_ST,1_ = 0 s (red line)

*Program:* To program the selected cell, the selected *V*_C_ decreases from 0.0 to − 0.8 V as shown in Table 1. This decreased *V*_C_ can facilitate the influx of carriers into the base region. As such, the selected cell is programmed with the *V*_GC_ of 0.4 V and *V*_AC_ of 1.4 V. Figure 6a shows the simulated energy band diagrams of the cell under programming at *T*_ST,0_ and *T*_ST,1_ = 10 s. The selected cell for the program operation can maintain the state-1 with low *H*_P_ even at the high *T*_ST,1_ of 10 s. However, the problem with the above approach is that all cells in the selected BL experience unwanted program operation by the *V*_GC_ of 0.4 V and *V*_AC_ of 1.4 V, which are larger than the *V*_GC_ of 0.4 V and *V*_AC,P_ of 1.2 V, respectively. To prevent this unwanted programming, *V*_G_ in all WLs except for the selected WL can be decreased from − 0.4 to − 1.2 V. In this way, the *V*_GC_ can be recovered back to − 0.4 V from 0.4 V. Figure 6b shows the simulated energy band diagrams of the unselected cells at *T*_ST,0_ and *T*_ST,1_ = 0 s. The unselected cells exhibit no change in energy band with the *V*_AC_ of 1.4 V if *V*_GC_ is below − 0.4 V. Thus, the selected cell exhibits the continuous state-1, while the unselected cells can avoid the unwanted program disturbance.

**Fig. 7:**
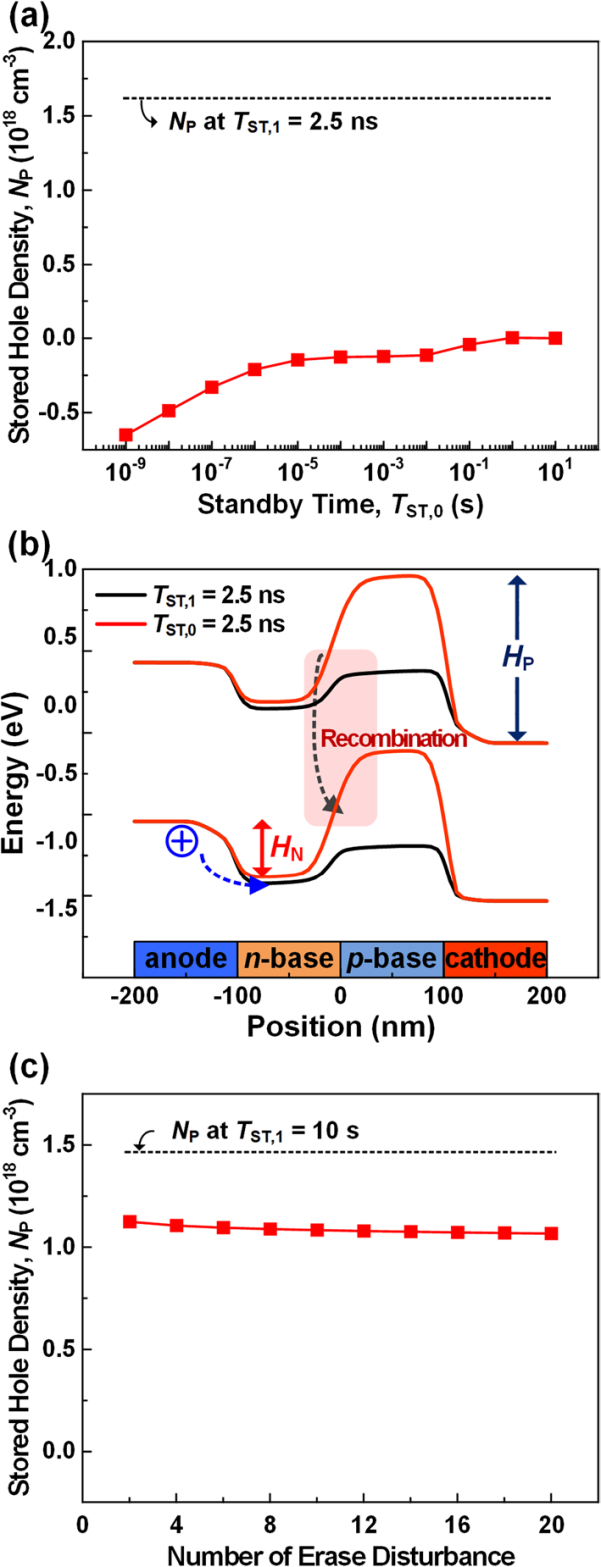
**a** *N*_P_ as a function of *T*_ST,0_ after the erase operation at *T*_ST,1_ = 2.5 ns. **b** Energy band diagrams at *T*_ST,1_ = 2.5 ns and *T*_ST,0_ = 2.5 ns. **c** *N*_P_ as a function of the number of the erase disturbance pulse

**Fig. 8:**
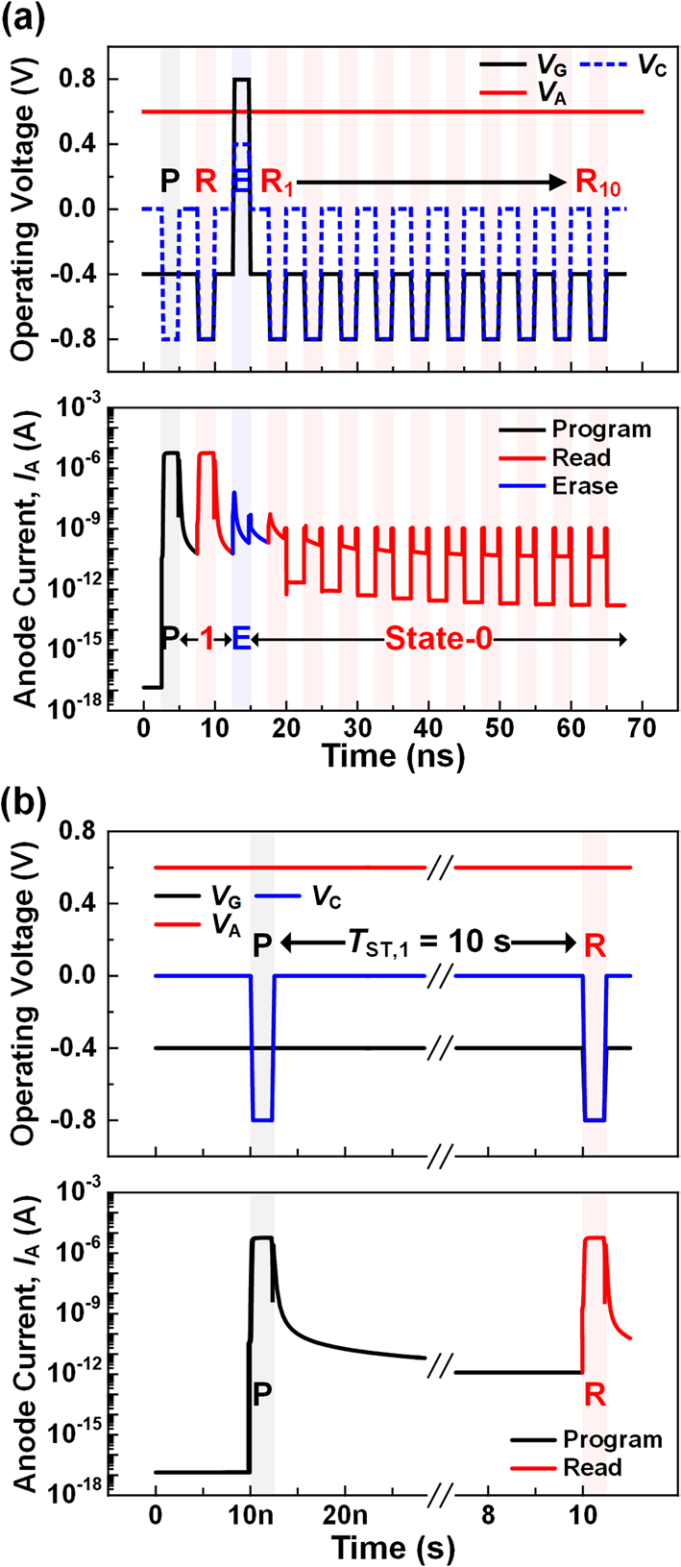
Operating voltage and anode current **a** when ten consecutive read operations are applied after the erase operation and **b** when a read operation is applied at *T*_ST,1_ = 10 s

*Erase:* To erase a selected cell, the *V*_G_ and *V*_C_ of the selected WL and BL should be increased from − 0.4 to 0.8 V and 0.0 to 0.4 V, respectively, as shown in Table 1. In Fig. 7a, the *N*_P_ is investigated as a function of *T*_ST,0_ after the erase operation at *T*_ST,1_ of 2.5 ns. The *N*_P_ is decreased due to the depletion of stored holes in the *p*-base as the *V*_GC_ increases from − 0.4 to 0.4 V. Also, the hole injection into the *p*-base during the erase operation is restrained by decreasing *V*_AC_ from 0.6 to 0.2 V. As the *T*_ST,0_ increases, the *N*_P_ is back to 0 cm^−3^, which represents the complete state-0. To investigate the reason, the energy band diagram at *T*_ST,0_ of 2.5 ns is examined and compares with the energy band at *T*_ST,1_ of 2.5 ns (Fig. 7b). After the erase operation, the *n*-base (*H*_N_) energy band height decreases as the *H*_P_ increases. The holes in the anode flow into the *p*-base over the lowered *H*_N_ and the *N*_P_ increase. The number of injected holes decreases due to the increased *H*_N_ by the recombination process, so the *N*_P_ saturates at 0 cm^−3^ as *T*_ST,0_ increases. From this result, it is found that the cell selected for the erase operation can exhibit the state-0 with the sufficiently high *H*_P_ at any *T*_ST,0_. However, the erasing method with the increased *V*_GC_ of the selected WL can cause a problem of erasing all cells on the same WL. To avoid this issue, the *V*_GC_ should be reduced from 0.4 to − 0.4 V by increasing *V*_C_ from 0.0 to 1.2 V on all BLs except for the selected BL (Table 1). Accordingly, the erase disturbance pulse with *V*_AC_ = − 0.6 V and *V*_GC_ = − 0.4 V applies to the unselected cells on the same WL. The state-1 should be detectable at any time even if this erase disturbance pulse is repeated after the *N*_P_ is saturated to the lowest value of 1.47 × 10^18^ cm^−3^. To confirm this, as shown in Fig. 7c, the *N*_P_ is examined as a function of the number of this erase disturbance pulse. Despite the repeated disturbance pulses, *N*_P_ exhibits a negligible decrease near 1.1 × 10^18^ cm^−3^ so that the device can maintain the state-1. In addition, the slightly reduced *N*_P_ can readily return to its original state-1 by applying read operation. Therefore, the 3-T TRAM can overcome the erase disturbance by controlling the *V*_GC_ in unselected cells,

*Parallel Read:* To perform the parallel read operations on the cells that share the same BL, the *V*_C_ of the selected BL and the *V*_G_ of all WLs are set to − 0.8 V (Table 1). If the *V*_C_ in the selected BL is decreased to − 0.8 V, not only *V*_AC_ increases to 1.4 V but also *V*_GC_ increases to 0.4 V. This high *V*_GC_ lowers the *H*_P_ and causes unwanted program errors of the cells in the selected BL. To avoid this, the *V*_G_ in all WLs should be decreased to − 0.8 V so that 0.0 V of *V*_GC_ and 1.4 V of *V*_AC_ are applied to the cells in the selected BL. To investigate the effect of the read operation on the state-0, the operating voltage and anode current are extracted after ten consecutive read operations are applied following an erase operation as shown in Fig. 8a. Although the ten continuous read pulses are applied to the device after the erase operation, the read current gradually decreases, confirming that the state-0 stably is maintained. This result indicates that the 3-T TRAM with the suggested array configuration for reading exhibits a reliable disturbance immunity for the state-0. Additionally, to confirm the detectability of the state-1, the operating voltage and anode current for a program and a read with the *T*_ST,1_ of 10 s are extracted (Fig. 8b). The read pulse can detect the state-1 continuously with a high current even at a long *T*_ST,1_ of 10 s.

## Conclusion

We have investigated the effects of the *V*_GC,ST_ and *V*_AC,ST_ of the nanoscaled 3-T TRAM for low-power operation and better retention characteristics. The optimized *V*_GC,ST_ of − 0.4 V allows a lower *V*_AC,P_ due to the accumulated holes in the standby state. In addition, a low *H*_P_ remains for a longer time because the optimized *V*_GC,ST_ effectively maintains the high *N*_P_ by reducing carrier recombinations at the junctions. The investigation of *I*_A_ − *V*_AC_ characteristics suggests that a minimum *V*_AC,ST_ of 0.6 V enables the device to exhibit the continuous state-1 without refresh operation while allowing a small standby current of 1.14 pA. Furthermore, a memory array operation strategy with the proper *V*_GC,ST_ and *V*_AC,ST_ for the 3-T TRAM is presented for the first time to implement reliable array operations without refresh and disturbances. The adjustment of *V*_GC_ can effectively minimize the program, erase and read disturbances in unselected cells. Along with the high immunity against array disturbances, the 3-T TRAM with the optimum strategy for array operations exhibits superior data retention capability than conventional 1T-1C DRAM technology. Thus, the proposed memory array operation scheme can provide a way to realize capacitorless 1T DRAM with 3-T TRAM.

## Data Availability

All data are fully available without restriction.

## Abbreviations

DRAM: Dynamic random-access memory
1T-1C: One transistor-one capacitor
3D: Three-dimensional
TRAM: Thyristor-based random-access memory
Si: Silicon
2-T: Two-terminal
3-T: Three-terminal
VCT: Vertical channel transistor
*V*_A_: Anode voltage
*V*_G_: Gate voltage
*V*_C_: Cathode voltage
*V*_GC_: Gate–cathode voltage
*V*_AC_: Anode–cathode voltage
*V*_GC,ST_: *V*_GC_ on the standby state
*V*_AC,ST_: *V*_AC_ on the standby state
*I*_A_: Anode current
WL: Word line
BL: Bit line
TCAD: Technology computer-aided design
*T*_rise_: Rise time
*T*_fall_: Fall time
*T*_hold_: Hold time
*V*_AC,P_: Program anode–cathode voltage
*H*_P_: Energy band barrier height in the *p*-base
*H*_N_: Energy band barrier height in the *n*-base
*N*_P_: Stored hole density in the *p*-base
*T*_ST,1_: Standby time at state-1
*T*_ST,0_: Standby time at state-0
VLSI: Very large-scale integration
